# Caregiver decision-making on pediatric research participation in congenital heart disease in western China: a qualitative study

**DOI:** 10.3389/fpubh.2026.1843389

**Published:** 2026-06-23

**Authors:** Mou Peng, Yuxin Ding, Zexi Li, Shuran Shao, Yaru Cui, Li Zhao, Yimin Hua, Kaiyu Zhou, Chuan Wang, Jinhui Li, Chunyi Yan

**Affiliations:** 1 Department of Pediatric Cardiology, West China Second University Hospital, Sichuan University, Chengdu, Sichuan, China; 2 Key Laboratory of Birth Defects and Related Diseases of Women and Children (Sichuan University), Ministry of Education, Chengdu, Sichuan, China; 3 Department of Obstetrics and Gynecology Nursing, West China Second University Hospital, Sichuan University, Chengdu, Sichuan, China; 4 Department of Cardiology, West China Hospital, Sichuan University, Chengdu, Sichuan, China; 5 Sichuan Provincial Children's Hospital, Meishan, Sichuan, China

**Keywords:** caregivers, clinical research participation, congenital heart disease, informed consent, therapeutic misconception, western China

## Abstract

**Background:**

High-quality pediatric clinical research depends on effective and ethically robust recruitment, yet participation can be difficult for families of children with congenital heart disease (CHD), particularly in resource-constrained and culturally diverse settings. In western China, long-distance care-seeking, financial strain, family-centered decision-making, and evolving pediatric research protections may shape how caregivers understand and negotiate research participation.

**Methods:**

We conducted a qualitative descriptive study at a tertiary pediatric referral center in western China between June and December 2025. Caregivers of children with CHD participated in one-to-one semi-structured interviews. Interviews explored practical burden, perceptions of research and treatment, therapeutic misconception, trust, child assent, and strategies to improve recruitment. Audio-recordings were transcribed verbatim, anonymized, and analyzed using qualitative content analysis.

**Results:**

Thematic saturation was reached after 22 interviews. Four overarching themes were identified: (1) families' real-world constraints and the costs of research participation; (2) cognitive biases and tensions surrounding therapeutic misconception; (3) trust anchors and views on assent, insurance, and institutional protection under a changing policy context; and (4) strategies for optimizing recruitment. Caregivers commonly weighed research participation against treatment-related burdens, especially travel distance, accommodation costs, wage loss, and repeated hospital visits. Decisions were further shaped by collective family decision-making, culturally mediated concerns about bodily integrity, confusion between research and individualized treatment, and strong reliance on physician recommendation. Participation was generally more acceptable when procedures were non-invasive or integrated into routine care, whereas extra venipuncture was often resisted. Caregivers also emphasized the value of plain-language, visual, dialect-adapted, and child-friendly communication.

**Conclusions:**

Caregivers' decisions about pediatric clinical research participation in western China are shaped by structural disadvantage, family-centered norms, therapeutic misconception, and trust in physicians and institutions. Recruitment should reduce burden, improve comprehension, support family communication, and accommodate children's developing role in research decisions.

## Introduction

Congenital heart disease (CHD) is among the most common congenital anomalies worldwide and remains a major contributor to childhood morbidity and mortality (1). In China, the expansion of newborn screening and sustained public health investment has improved the early detection of CHD, yet these advances have not been distributed evenly across regions (2, 3). This unevenness is especially evident in western China, where high-altitude environments, relatively limited socioeconomic development, and weaker primary healthcare capacity converge to produce a more challenging landscape for the detection and management of pediatric heart disease (4). The public health relevance of this disparity is closely aligned with the *Healthy China 2030* agenda, which explicitly emphasizes health equity, accessibility, and the reduction of regional gaps in health services (5). However, despite these national goals, western China continues to experience substantial inequities in healthcare resource allocation and service accessibility (2, 6). For children with CHD, these structural disadvantages are not abstract system-level problems; they translate into delayed diagnosis, prolonged care-seeking trajectories, and reduced opportunities to benefit from specialized pediatric cardiac care.

These constraints also shape the realities of clinical research participation. Pediatric cardiac services in less-developed regions are typically concentrated in a limited number of tertiary referral centers, meaning that many families must travel long distances across counties or provinces for diagnosis, surgery, and follow-up (3, 7). For socioeconomically vulnerable households, the cumulative burden of transportation, accommodation, wage loss, and repeated hospital visits can be substantial, and these burdens may directly undermine the feasibility of longitudinal research participation (8–10). This issue is particularly important in pediatric medicine, where high-quality clinical research is essential for improving evidence-based treatment and advancing drug development, yet participant recruitment remains difficult worldwide (10, 11). In western China, the situation is further complicated by the region's multi-ethnic and culturally diverse population, including Tibetan and Yi communities, in which medical decisions are often embedded in family-centered, relational, and culturally mediated norms rather than made solely by one parent acting independently (12, 13). In such contexts, decisions about a child's body and treatment are shaped not only by practical barriers, but also by family hierarchy, locally grounded understandings of illness, and strong deference to professional authority.

A further challenge is therapeutic misconception (TM), a well-recognized ethical problem in clinical research. TM arises when participants or proxy decision-makers do not adequately distinguish the scientific purpose of research from the individualized goal of treatment (14–16). For caregivers of children with CHD, especially those confronting serious illness, geographic isolation, and limited health literacy, this distinction may be particularly difficult to sustain. Standardized trial procedures, frequent contact with research staff, and recommendations from senior specialists at high-level centers may be interpreted as signs of preferential or personalized treatment rather than as elements of a protocol designed to generate generalizable knowledge (14). This issue has become even more salient in China's evolving regulatory environment. Recently released Chinese guidance for pediatric drug clinical trials emphasizes scientific necessity, maximal benefit, risk minimization, and assent procedures tailored to children and guardians (17). Although this guidance specifically addresses pediatric drug trials, it reflects a broader strengthening of pediatric research protections in China. While these developments strengthen participant protection and acknowledge the developing autonomy of minors, they may also create new tensions in western China, where family-centered decision-making remains highly influential.

The health belief model (HBM) may offer a useful interpretive lens for understanding some aspects of these decisions, particularly perceived barriers, expected benefits, and cues to action (18, 19). However, caregiver decision-making in this context is also shaped by broader structural, cultural, and relational factors that may not be fully captured by any single behavioral model (13). Therefore, the present qualitative study explores how caregivers of children with CHD in western China understand and negotiate research participation, while drawing on the HBM as a sensitizing rather than prescriptive framework. By clarifying how these factors intersect in a resource-constrained and culturally diverse region, this study aims to inform more context-sensitive, ethically robust, and equitable strategies for recruitment, communication, and informed consent in pediatric clinical research.

## Methods

### Study design

This study used a qualitative descriptive design to explore how caregivers of children with CHD in western China understood and negotiated decisions about participation in clinical research. Reporting was informed by the consolidated criteria for reporting qualitative research (COREQ) (20). The study was conducted between June 2025 and December 2025 at a tertiary pediatric referral center in western China.

### Research team and reflexivity

The interviews were conducted by three researchers from the pediatric cardiology team, including one female nurse and two male physicians, all of whom had received prior training in qualitative interviewing. The interviewers were involved in pediatric clinical research but had no prior clinical or personal relationship with the participants. Before data collection and during analysis, the research team discussed their assumptions regarding caregiver decision-making, TM, trust in physicians and institutions, and family-centered consent in western China in order to reduce premature interpretation. Reflexive notes were maintained during interviewing and coding to document interviewer impressions, emotional responses, and evolving analytic assumptions.

### Study setting and participants

The study was conducted at a national-level regional pediatric medical center in western China. The participant pool consisted of caregivers who were accompanying children admitted to the inpatient pediatric cardiology ward during the study period. This study did not identify eligible families through a retrospective medical-record query, nor were families called back to the hospital solely for study participation. Participants were recruited using purposive maximum-variation sampling to capture a broad range of caregiver perspectives. Sampling sought variation in caregiver identity, ethnicity, place of residence, travel burden, payment method, and household economic circumstances. Parental educational level was not used as a formal stratification variable; however, residence type, occupation, household income, and ethnicity were considered during sampling because these factors partly reflected socioeconomic and informational diversity in this regional context. Eligible participants were the primary legal guardian or a long-term caregiver of a child diagnosed with CHD, were able to communicate in Mandarin, and were willing to provide written informed consent. Mandarin is the official language used for hospital-based communication, while Sichuanese/Southwestern Mandarin is commonly spoken in the local area. Some families from remote minority regions may also use Tibetan, Yi, or other minority languages in family or community settings. In this study, references to dialect- or language-adapted communication therefore concern accessibility and cultural appropriateness rather than socioeconomic status. Caregivers of children in an acute life-threatening condition or requiring emergency resuscitation were excluded so that participation could be considered voluntarily and with adequate deliberation. A total of 22 caregivers were interviewed, including mothers, fathers, and one grandmother, with marked diversity in ethnicity, residence type, travel time, and insurance coverage. Recruitment continued until thematic sufficiency was judged to have been reached, that is, when successive interviews no longer generated substantially new codes, categories, or interpretive dimensions relevant to the study aim (21, 22). During the recruitment period, 22 eligible caregivers were approached, all agreed to participate, and all completed the interview.

### Data collection

Data were collected through semi-structured, in-depth interviews. Each caregiver was invited to choose a convenient interview time. Interviews were conducted in a private consultation room within the inpatient ward, lasted approximately 45 min on average, and were audio-recorded with permission. Field notes were taken during and immediately after each interview. Audio files were transcribed verbatim within 24 h and anonymized using participant codes (P01–P22). No repeat interviews were conducted. Sampling ended when the authors discussed that the data reached a saturation point.

The interview guide was developed through literature review, discussion within the research team, and consultation with clinical experts (10, 23), and the final version is provided as Supplementary material 1. The guide was designed broadly enough to capture issues extending beyond any single behavioral model. It covered four main areas: (1) care-seeking context, information sources, and practical burden;(2) perceptions of clinical research, including perceived benefits, burdens, and possible TM; (3) family decision-making, trust, privacy, child assent, and insurance-related protection; and (4) strategies for improving recruitment and communication for families from remote or minority regions. Some prompts were informed by behavioral concepts such as perceived barriers, expected benefits, and cues to action, but the guide was not structured as a formal HBM instrument. Neutral probes such as “Could you tell me more about that?” and “Can you give an example?” were used to clarify meaning and deepen responses.

Because not all caregivers had previous experience with research recruitment, a brief hypothetical scenario was used when necessary to introduce the general idea of pediatric clinical research before asking procedure-related questions. The scenario emphasized voluntariness, non-interference with treatment, optional participation in procedures of varying invasiveness, confidentiality, and the possibility of withdrawal without affecting care. It was used only to facilitate understanding and was not intended to standardize or predetermine participants' responses. Procedure-related questions explored gradients of acceptability, including questionnaire-based or observational participation, add-on sampling during routine blood collection, mildly uncomfortable procedures such as swabs, and extra venipuncture performed solely for research. Additional questions examined caregivers' views on family disagreement, timing of consent discussions, privacy and data use, return of research-related information, the role of physician endorsement, and communication formats that might reduce decisional burden, such as plain-language summaries, visual diagrams, or dialect-adapted materials.

### Data analysis

Data analysis was conducted concurrently with data collection using qualitative content analysis as described by Graneheim and Lundman (24). Analysis focused on both manifest content and the abstraction of latent meaning. First, transcripts were read repeatedly to obtain an overall sense of the material. Second, text segments relevant to the study aim were identified as meaning units and condensed while preserving their core meaning. Third, condensed meaning units were assigned initial codes. Fourth, similar codes were compared and grouped into subcategories and broader categories. Finally, overarching themes were developed through iterative abstraction across interviews. ATLAS.ti 23 was used to support data management, coding, and retrieval.

### Rigor

Several strategies were used to enhance rigor. First, the interviewers had prior training in qualitative interviewing. Second, interviews were conducted in a private setting, and interviewers maintained a neutral, non-leading stance. Ambiguous or emotionally charged statements were clarified in real time. Third, analyst triangulation was applied: two researchers independently reviewed transcripts and codes, and disagreements were resolved through discussion with a third researcher. These contextual interviews were used only to support background understanding and were not included in the formal thematic analysis of caregiver interviews. Finally, participants were contacted by telephone to verify the accuracy of key transcript content and the intended meaning of selected statements, and clarifications were incorporated where necessary. All interviews were conducted in Chinese. Quotations presented in English were first translated by the original interviewer and were then checked against the Chinese transcripts by two senior pediatric cardiovascular clinicians who had not conducted the interview, including one physician and one nurse, to preserve conceptual meaning and contextual nuance, and minor edits were made only for readability.

### Ethical considerations

Ethical approval was obtained from the Ethics Committee of the participating hospital (Approval No. 2025-007), and written informed consent was obtained from all participants before interview. Participants were informed that participation was voluntary, that they could refuse to answer any question, and that refusal or withdrawal would not affect the child's clinical care. No minors were directly interviewed in this study. Because the interview guide included questions about caregivers' views on child assent, institutional protection, and research-related risk, interviews were conducted with attention to participants' emotional state and willingness to continue. All transcripts were de-identified before analysis, and only the research team had access to the study data.

## Results

### Demographic characteristics

After discussion among the authors, thematic sufficiency was judged to have been reached by the 22nd interview. The demographic and clinical characteristics of the 22 participating caregivers and their children are summarized in Table 1. Thus, 22 primary caregivers of children with CHD participated in the study, including 16 mothers, 5 fathers, and 1 grandmother. The sample included Han (54.5%), Tibetan (22.7%), Yi (13.6%), Qiang (4.5%), and Bouyei (4.5%) caregivers. Children ranged in age from 6 months to 15 years, with boys slightly outnumbering girls (13 vs. 9).

**Table 1:** Characteristics of caregivers and children, research participation attitudes, and therapeutic misconception profiles (*N* = 22).

ID	Identity	Ethnicity	Occupation^*^	Residence type^†^	Monthly household income (RMB)^‡^	Travel time	Payment method	Child age/sex	General willingness	Attitude toward extra blood draw	Therapeutic misconception (TM)^§^
P01	Mother	Han	Salaried employee	Urban district	5,000–9,999	< 2 h	UEBMI	5 y/M	Willing	Refused (fear of pain)	None
P02	Mother	Tibetan	Homemaker	Rural/remote area	< 5,000	>6 h	URRBMI	9 y/F	Willing	Hesitant (follow physician advice)	Goal misconception
P03	Father	Han	Self-employed	County town	5,000–9,999	2–6 h	URRBMI	12 y/M	Willing	Acceptable (add-on sampling)	None
P04	Mother	Yi	Homemaker	Rural/remote area	< 5,000	>6 h	URRBMI	4 y/F	Hesitant	Refused	Procedural misconception
P05	Mother	Han	Public-sector worker	Urban district	10,000–14,999	< 2 h	UEBMI	11 y/M	Willing	Acceptable (add-on sampling)	None
P06	Mother	Tibetan	Homemaker	Rural/remote area	< 5,000	>6 h	URRBMI	6 y/F	Willing	Acceptable (follow senior doctor)	Goal misconception
P07	Father	Yi	Farmer	Rural/remote area	< 5,000	>6 h	URRBMI	8 y/M	Refused	Strongly refused	Cultural sensitivity
P08	Mother	Han	Homemaker	County town	5,000–9,999	2–6 h	URRBMI	3 y/M	Willing	Refused (fear of harm)	None
P09	Mother	Qiang	Homemaker	Rural/remote area	< 5,000	>6 h	URRBMI	14 y/F	Willing	Acceptable (add-on sampling)	None
P10	Mother	Han	Homemaker	Urban district	5,000–9,999	< 2 h	UEBMI	2 y/M	Hesitant	Refused	Fear-based response
P11	Grand-mother	Han	Farmer	County town	< 5,000	2–6 h	URRBMI	10 y/M	Willing	Acceptable (authority-dependent)	Goal misconception
P12	Mother	Bouyei	Small vendor	Rural/remote area	< 5,000	>6 h	URRBMI	5 y/F	Willing	Acceptable (altruistic motive)	Research = treatment
P13	Father	Han	Public-sector worker	Urban district	≥15,000	< 2 h	Commercial insurance	13 y/F	Willing	Hesitant (depends on purpose)	None
P14	Mother	Tibetan	Manual laborer	Rural/remote area	< 5,000	>6 h	URRBMI	7 y/M	Willing	Hesitant	Expected economic gain
P15	Mother	Han	Self-employed	County town	5,000–9,999	2–6 h	UEBMI	9 y/F	Willing	Refused	Protection-based avoidance
P16	Mother	Yi	Farmer	Rural/remote area	< 5,000	>6 h	URRBMI	4 y/M	Refused	Strongly refused	Sees research as experimentation
P17	Father	Tibetan	Herdsman	Rural/remote area	< 5,000	>6 h	URRBMI	15 y/M	Willing	Acceptable (add-on sampling)	None
P18	Mother	Han	Homemaker	Urban district	5,000–9,999	< 2 h	UEBMI	6 mo/F	Hesitant	Refused	Severe preoperative anxiety
P19	Mother	Han	Self-employed	County town	5,000–9,999	2–6 h	URRBMI	11 y/M	Willing	Acceptable (altruistic motive)	None
P20	Mother	Tibetan	Homemaker	Rural/remote area	< 5,000	>6 h	URRBMI	3 y/M	Willing	Acceptable (authority-dependent)	Goal misconception
P21	Father	Han	Salaried employee	Urban district	10,000–14,999	< 2 h	UEBMI	8 y/F	Willing	Hesitant	None
P22	Mother	Han	Self-employed	County town	5,000–9,999	2–6 h	URRBMI	12 y/M	Willing	Acceptable (add-on sampling)	None

UEBMI, urban employee basic medical insurance; URRBMI, urban and rural resident basic medical insurance.

^*^Occupation refers to the caregiver's primary employment status or main source of household livelihood.

^†^Residence type refers to the family's usual place of residence before referral to the study center.

^‡^Monthly household income is presented as self-reported total household income before major CHD-related medical expenditure. (7 RMB ≈ 1 USD, therefore 5,000 RMB ≈ 700 USD)

^§^The TM column is presented here as a qualitative profiling field rather than a formal psychometric classification.

Overall, the sample reflected marked socioeconomic and geographic vulnerability. Homemakers were the largest occupational subgroup (36.4%), and half of the families reported a monthly household income below Chinese yuan (RMB) 5,000. Nearly half of participants lived in rural or remote pastoral areas (45.5%), and the same proportion reported a one-way travel time of more than 6 h to the study center. Most families relied on Urban and Rural Resident Basic Medical Insurance (URRBMI) (68.2%), whereas only one reported commercial supplementary insurance. These characteristics indicate that many participating caregivers were making research-related decisions under conditions of limited resources and substantial care-seeking burden. The travel time and cost described by participants referred primarily to the burden of seeking specialist diagnosis, admission, treatment, and follow-up for the child's CHD, rather than additional travel required for participation in this interview study.

### Qualitative analysis of caregivers' in-depth interviews

Although the interview guide was informed in part by the HBM, data analysis was primarily inductive rather than theory-driven. Coding was not organized around predefined HBM constructs. Instead, the HBM functioned only as a sensitizing framework during study design and later interpretation, while the analytic structure was allowed to emerge from participants' narratives through constant comparison across interviews (15). This process yielded four overarching themes, 15 main categories, and related subcategories, as presented in Table 2.

**Table 2:** Themes, main categories, and subcategories derived from qualitative interviews with caregivers of children with congenital heart disease in western China.

Themes	Main categories	Subcategories
Families' real-world constraints and the costs of research participation	Travel and financial burden	Long-distance referral burden. Wage loss and accommodation costs. Financial sensitivity to additional follow-up. Preference for combining research with routine visits.
	Family-cultural decision context	Collective family decision-making. Elders' influence on consent. Cultural concerns about blood and bodily integrity. Fear of community stigma and gossip.
	Information and understanding barriers	Information overload during digital health-seeking. Difficulty identifying trustworthy sources. Poor understanding of research terminology. Reliance on physician authority when comprehension is limited.
Cognitive biases and tensions surrounding therapeutic misconception	Research-treatment confusion	Research interpreted as personalized treatment. Confusion between clinical care and study procedures. Research participation perceived as intensified medical attention.
	Procedure acceptability gradient	Acceptance of non-invasive participation; conditional acceptance of add-on sampling. Resistance to extra venipuncture. Perceived difference between routine care and “extra” procedures.
	Prior medical trauma	Defensive refusal after repeated painful procedures. ICU- and surgery-related anxiety. Heightened sensitivity to optional interventions.
	Overestimation of benefit	Expectation of closer physician monitoring. Perceived privileged access to care. Assumption of greater benefit from participation.
	Science-family needs mismatch	Priority given to immediate burden over future benefit. Tension between altruism and self-interest. Limited value attached to abstract knowledge generation.
Trust anchors and responses to the 2025 regulatory changes	Digital and physician trust	Trust in official hospital communication channels. Strong dependence on physician recommendation. Written consent viewed as less influential than verbal endorsement.
	Child assent responses	Recognition of children's developing autonomy. Concern about child refusal due to fear. Potential conflict between family authority and child assent.
	Insurance and institutional protection	Reassurance from explicit compensation clauses. Trust strengthened by trial insurance disclosure. Institutional prestige as a proxy for safety and legitimacy.
Strategies for optimizing recruitment	Visual plain-language communication	Use of pathway diagrams to distinguish treatment from research. Simplified explanations of study procedures. Preference for less text-heavy consent communication.
	Dialect-adapted communication	Dialect- or minority-language educational materials. Culturally adapted communication. Improved accessibility for remote families.
	Clinical pathway integration	Aligning study follow-up with routine clinical visits. Minimizing extra hospital trips. Preference for add-on rather than separate procedures.
	Child-friendly assent support	Cartoon-style or illustrated assent materials. Developmentally appropriate explanations for older children. Parent–child joint discussion during assent.

The qualitative analysis of caregivers' in-depth interviews is presented in Table 2. Four overarching themes were identified: (1) families' real-world constraints and the costs of research participation; (2) cognitive biases and tensions surrounding TM; (3) trust anchors and responses to the 2025 regulatory changes; and (4) strategies for optimizing recruitment. The following text presents the main categories derived from the data. All the following quotes are taken directly from the patients' original words, with the patient number and ethnicity in parentheses.

### Theme 1. Families' real-world constraints and the costs of research participation

Three main categories described the real-world context that shaped caregivers' decisions about research participation: long travel distance and financial hardship, culturally embedded decision-making in a multi-ethnic context, and barriers to information access coupled with variability in understanding. Across interviews, caregivers consistently framed participation not as a separate research decision, but as something weighed against the burdens already associated with caring for a child with CHD and traveling for treatment.

#### Long travel distance and financial hardship

Long travel distance and financial hardship were repeatedly described as major barriers to research participation. For many caregivers, the cost of treatment was not limited to hospital bills, but also included transportation, accommodation, wage loss, and the physical strain of repeated long-distance travel. Research was often viewed as acceptable only when it could be integrated into routine follow-up.

“*We came from Liangshan Prefecture … If participating in the study means we have to stay in the hospital longer or make a special trip back just for follow-up, the little money we have saved really cannot sustain that … The cost of travel and accommodation is even higher than the examination fee itself.” (P16, Yi)*

“*It is just too far … If it can be arranged on the same day as the postoperative follow-up, then we would sign. But if we have to make a separate trip just for the research, no amount of subsidy would truly cover our travel costs and lost time …” (P02, Tibetan)*

“*Although the hospitalization expenses can be reimbursed … our family has already borrowed fifty to sixty thousand yuan … We are extremely sensitive to any additional expense …” (P14, Tibetan)*

#### Culturally embedded decision-making in a multi-ethnic context

Decisions about research participation were often shaped by collective family decision-making and culturally grounded beliefs about the child's body. In several rural and minority families, the interviewed caregiver did not see herself or himself as the sole decision-maker. Concerns about blood drawing, bodily integrity, and the social meaning of participating in “experiments” were especially prominent.

“*The elders in our family heard that extra blood would be drawn … and said that this would ‘disturb the child's vital essence' … if the elders do not agree, I would not dare sign those papers on my own.” (P07, Yi)*

“*Signing this kind of document has to be discussed by the whole family … If they think this kind of research might ‘disturb' the child's good fortune, then we cannot sign.” (P11, grandmother)*

“*Our hometown is a small place. If people found out that the child had participated in some kind of experiment … they would gossip … We do not want other people to know about it.” (P12, Bouyei)*

#### Barriers to information access coupled with variability in understanding

A third barrier concerned the burden of obtaining and interpreting health information. Caregivers often used several information sources, including hospital public accounts, online parent groups, and social media platforms. However, access to more information did not always improve understanding. Instead, inconsistent explanations often generated confusion, especially when caregivers tried to distinguish routine treatment information from research-related information.

“*I usually search online first, and I also read the hospital's public account, but honestly, the more I read, the more confused I become … I cannot tell which information is really reliable.” (P19, Han)*

“*When the doctor talks about treatment, I can more or less follow. But once they start using terms like ‘random assignment' or ‘control group,' I get lost … if the doctor says it is okay, then maybe I should trust the doctor.” (P20, Tibetan)*

### Theme 2. Cognitive biases and tensions surrounding TM

Five main categories described how caregivers interpreted the meaning, risks, and potential value of research participation: confusion between therapeutic goals and research procedures, a gradient of acceptability toward different procedures, the impact of prior medical trauma, therapeutic overestimation bias, and a disconnect between scientific logic and families' practical needs.

#### Confusion between therapeutic goals and research procedures

Some caregivers confused the purpose of research with individualized treatment. Research procedures were sometimes interpreted as a special treatment arrangement for the child rather than as part of a standardized study.

“*I thought that the ‘random assignment' … must mean that the department director … would place us in the group with the best medical resources … As long as it is a study introduced by specialists … it must be more advanced than ordinary treatment.” (P06, Tibetan)*

“*I signed that form because I believed a research project would definitely involve more careful assessment … I felt that my child was receiving better care. Isn't that specifically for treating my child?” (P20, Tibetan)*

#### A gradient of acceptability toward different procedures

Caregivers showed different levels of acceptance toward different research procedures. Non-invasive activities and add-on sampling during routine blood collection were generally more acceptable, whereas extra venipuncture for research alone was often resisted.

“*If it is only asking questions … while we are already here, then I think that is okay. But if the child has to be punctured again only for the study, then that becomes a different matter.” (P03, Han)*

“*If the blood can be taken together with the tests the child already needs … we might accept it. But if it means another needle just because the researchers need it, I would hesitate immediately.” (P15, Han)*

“*Doing it together with the regular examination is one thing. Doing it separately just for research feels different …” (P08, Han)*

#### The impact of prior medical trauma

Previous painful or stressful medical experiences increased caregivers' reluctance toward additional research-related procedures. For some families, the child's accumulated treatment burden made even minor extra procedures difficult to accept.

“*They asked me to use that ‘pulmonary function machine,' and I assumed it was … a necessary postoperative examination. I never thought that it was only for collecting data for their research.” (P09, Qiang)*

“*The child has already suffered so much in the hospital … If something is not directly for treatment, my first reaction is to refuse …” (P18, Han)*

“*After surgery, the child becomes very nervous as soon as anyone in a white coat comes close … I still feel resistant.” (P10, Han)*

#### Therapeutic overestimation bias

Some caregivers overestimated the direct personal benefit of participation and associated research with closer monitoring or better access to treatment.

“*If the hospital includes us in a study, I would feel that the doctors are paying more attention to my child … Maybe that means we will get a better chance at treatment …” (P06, Tibetan)*

“*If joining the study means the doctors will observe the child more closely … then I would naturally think that participation is beneficial.” (P13, Han)*

“*When a child is in a study, I feel the doctors won't just treat it casually …” (P21, Han)*

#### A disconnect between scientific logic and families' practical needs

Caregivers often evaluated research in terms of immediate family burden rather than long-term scientific value. Altruistic reasoning was present, but it was often secondary to present concerns about pain, time, travel, and cost.

“*I understand that research can help other children in the future … But … I still have to think first about whether it adds trouble for my child and for our family right now.” (P19, Han)*

“*If it does not directly help my child, then I have to think very carefully about why we should take on extra cost, extra time, and extra risk …” (P14, Tibetan)*

“*Helping medical research sounds good, but … the first question is still whether the child has to suffer more or whether we have to spend more.” (P17, Tibetan)*

### Theme 3. Trust anchors and views on assent, insurance, and institutional protection under a changing policy context

#### Digital authority and physician endorsement as dual trust anchors

Trust emerged as a decisive cue to action. Many caregivers described a dual trust structure in which official hospital channels provided reassurance, while endorsement from the treating physician remained the most influential factor. In practice, a trusted clinician's recommendation could outweigh lengthy written materials.

“*I would first look at the hospital's official account, because at least that feels more formal than what people post in group chats.” (P19, Han)*

“*When the attending doctor explains it face to face, I feel much more reassured... If the doctor says this study is safe and meaningful, I am more likely to believe it.” (P21, Han)*

#### Responses to the child assent requirement for those aged 8 years and older

Caregivers showed mixed responses to the requirement that children aged 8 years and older should also provide assent. Some regarded this as reasonable and respectful, whereas others worried that it might create tension if an older child refused because of fear or discomfort.

“*It is reasonable to ask older children what they think, but if my child refuses only because he is afraid of a needle, then as a parent I would feel very conflicted.” (P15, Han)*

“*If the child is already old enough to understand a little, then asking for his opinion is fair. But in the end, parents still have to think about what is best.” (P13, Han)*

#### Perceived protection through insurance, compensation, and institutional legitimacy

Explicit information about insurance and compensation was widely interpreted as a sign of institutional seriousness. Institutional reputation also reduced uncertainty, and studies linked to a national or regional referral center were perceived as more trustworthy.

“*Only when I saw the compensation clause written clearly did I feel that we were actually protected.” (P15, Han)*

“*If our local hospital (county or city hospital) invited us to participate in the research, we might not agree.” (P02, Tibetan)*

“*Because this is a regional central hospital and also a national-level children's medical center, we are not worried about these issues. ” (P04, Yi)*

### Theme 4. Strategies for optimizing recruitment

#### Visual and plain-language communication

Caregivers repeatedly suggested that recruitment materials should be simplified and made more visual. Many felt that the distinction between treatment and research would be easier to understand if presented through diagrams, comparison charts, or short plain-language materials.

“*If there were a simple chart showing what belongs to treatment and what belongs to research, it would be much easier for us to understand than just reading pages of text.” (P12, Bouyei)*

“*Sometimes the consent form is too long. A shorter explanation with pictures would help us understand the key points faster.” (P19, Han)*

#### Dialect- and context-adapted communication for remote and minority families

Several caregivers indicated that standard written explanations might not be sufficient for families from remote or ethnically diverse areas. Dialect-based or more locally adapted communication was seen as more practical than formal text alone.

“*For families from remote minority areas, a plain-language or dialect video would be more useful than a long written explanation.” (P16, Yi)*

“*Some older family members cannot follow the formal language very well, so if the explanation were simpler, it would make discussion at home easier.” (P11, grandmother)*

#### Integration into the clinical pathway to reduce burden

Caregivers emphasized that research would be more acceptable if procedures were embedded into existing clinical care. Synchronizing research activities with routine follow-up and prioritizing add-on sampling over extra punctures were seen as practical ways to reduce burden.

“*If it can be arranged together with the routine follow-up, then it feels manageable. The hardest part is making a separate trip only for research.” (P02, Tibetan)*

“*Doing the research together with the regular examination makes sense. What we are afraid of is extra visits, extra waiting, and extra procedures.” (P08, Han)*

#### Child-friendly assent materials and family-centered communication

Under the new policy environment, caregivers suggested that assent for older children should not rely solely on adult-oriented documents. Child-friendly explanations and parent–child joint communication were viewed as more feasible and humane.

“*For older children, maybe a cartoon-style explanation or a simpler page would work better. A child will not understand the same form that adults read.” (P19, Han)*

“*If the child has to sign too, then someone should explain it in a way the child can actually understand, not just hand over the adult form.” (P21, Han)*

## Discussion

This study explored how caregivers of children with CHD in western China make decisions about participation in clinical research. Guided by HBM, the findings suggest that these decisions are shaped by the interaction of structural barriers, family-centered cultural norms, TM, and trust in physicians and institutions. Rather than being treated as an isolated research choice, participation was embedded in the broader realities of long-distance care-seeking, unequal access to specialist services, and financial vulnerability in western China.

A major finding was that geographic and economic constraints strongly influenced willingness to participate. Many caregivers described participation as feasible only when it could be incorporated into routine clinical follow-up, whereas an additional trip, extra hospitalization time, or further out-of-pocket spending substantially reduced acceptability. This finding is consistent with evidence showing persistent regional inequities in healthcare resource allocation in China and reduced medical convenience in ethnic minority areas of Southwest China (4, 6, 25). It also aligns with previous qualitative work indicating that parental decisions about pediatric research are closely tied to the practical burden of travel, time, and household finances (26). In our study, these material constraints were further embedded in family-centered decision-making, which supports prior scholarship suggesting that informed consent in China is often negotiated within relational and intergenerational family structures rather than exercised as a purely individual act.

Another important finding was the persistence of TM. Some caregivers interpreted standardized research procedures as personalized treatment or as a route to closer physician attention and better access to care, which is consistent with the broader literature on TM in clinical research (14, 27). In this study, however, TM appeared to be reinforced by the local context: caregivers were making decisions in a setting marked by disease severity, limited health literacy, and strong dependence on specialist authority. This suggests that TM should not be understood only as an individual cognitive problem, but also as a context-sensitive phenomenon shaped by scarcity, trust, and unequal access to high-quality care (14). The findings also showed a clear procedural gradient: non-invasive participation and add-on blood sampling were more acceptable, whereas extra venipuncture for research alone was often resisted (28–30). This pattern indicates that caregivers evaluated research not in abstract methodological terms, but in relation to anticipated pain, prior medical trauma, and whether a procedure appeared clinically meaningful for their own child.

Trust functioned as a major cue to action. Caregivers described a dual trust structure in which official hospital communication channels provided reassurance, while direct endorsement from the treating physician remained the strongest influence on decision-making. This finding is consistent with the Chinese doctor-family-patient model and with evidence on family roles in informed consent (12, 31). It also suggests that institutional digital communication may serve as a useful trust infrastructure in contexts where caregivers must navigate fragmented or inconsistent online information. At the same time, the 2025 pediatric trial guidance has made these issues more complex. The requirement that children aged 8 years and older should provide assent in addition to guardian consent strengthens participant protection, but our findings suggest that it may also generate tension when a child's refusal conflicts with family authority or treatment expectations. This tension is compatible with broader discussions on assent, child autonomy, and the challenge of reconciling developing child rights with Confucian family ethics in China.

Taken together, the present findings suggest that the HBM was useful primarily as a partial interpretive lens rather than a complete explanatory framework. Caregivers' narratives clearly reflected perceived barriers, expected benefits, and cues to action. However, they also highlighted TM, relational trust and family hierarchy, which extend beyond the classic HBM constructs. This suggests that decisions about pediatric research participation in this setting are shaped not only by individual health beliefs, but also by structural disadvantage, institutional context, and family-centered moral reasoning.

These findings have practical implications for pediatric research recruitment. First, recruitment strategies should reduce avoidable burden by aligning study procedures with routine clinical pathways wherever possible, especially for families traveling long distances. Second, the consent process should be family-oriented in practice while still maintaining clear communication with the primary caregiver and, when appropriate, with the child. Third, communication materials should be simplified and diversified through visual pathway maps, plain-language explanations, dialect-adapted materials, and child-friendly assent tools (32, 33). Importantly, these materials should explicitly distinguish research participation from individualized clinical treatment, clarify which procedures are required for routine care and which are conducted solely for research, and avoid implying that enrollment will necessarily provide direct therapeutic benefit. Such communication may help reduce therapeutic misconception while supporting more informed and voluntary decision-making. Finally, explicit disclosure of insurance, compensation mechanisms, and institutional oversight should be used not only to satisfy regulatory requirements but also to strengthen trust and reduce perceived risk. Although the specific findings should not be assumed to be directly transferable to all pediatric cardiology settings, the underlying themes may have broader relevance. Families' decisions about pediatric research participation are shaped not only by distance and cost, but also by trust, perceived benefit, prior medical experiences, family decision-making, communication barriers, and concerns about research-related procedures. These factors may be overlooked unless research teams actively seek to understand family perspectives before designing recruitment and consent processes.

This study has several limitations. First, as a single-center qualitative study conducted at a high-level tertiary referral hospital, the findings are intended to provide contextual and interpretive understanding rather than statistical generalization. Participants may also have expressed greater deference to specialist authority than would be seen in lower-level or community settings. Second, the study included caregivers of clinically stable children with CHD and did not include children whose condition was life-threatening at the time of recruitment; therefore, the findings may not fully capture the perspectives of families facing severe, unstable, or highly complex CHD. Third, parental educational level was not used as a formal sampling stratum, although sampling sought variation in residence type, ethnicity, occupation, income, and travel burden. Fourth, participants were limited to caregivers who could communicate in Mandarin, which may underrepresent families with greater language barriers. Finally, the study examined caregivers' stated views rather than real-time enrollment decisions in an active trial, and children themselves were not directly interviewed.

In conclusion, caregivers' decisions about pediatric research participation in western China are shaped by structural disadvantage, culturally embedded family decision-making, TM, and evolving regulatory expectations. Improving recruitment in this setting will require more than standard written consent. It will require a context-sensitive approach that reduces practical burden, improves comprehension, supports family communication, and respects the developing role of children in research decisions.

## Data Availability

The raw data supporting the conclusions of this article will be made available by the authors, without undue reservation.
