# Supplementary material for: Caregiver decision-making on pediatric research participation in congenital heart disease in western China: a qualitative study
Source: Front Public Health. 2026 Jun 23;14:1843389. doi: 10.3389/fpubh.2026.1843389 (PMC13340412; doi:10.3389/fpubh.2026.1843389)
Supplement: Supplementary file 1 [file Data_Sheet_1.docx]

**Semi-structured interview guide**

**Purpose.** This guide was developed to explore how caregivers of children with congenital heart disease make decisions about participation in clinical research in western China. The guide aligns with the study’s analytic focus on structural barriers, therapeutic misconception, trust, child assent, and strategies to optimize recruitment.

# **General instructions for interviewers**

**•** Begin by introducing the study, obtaining verbal agreement to proceed, and confirming permission for audio recording. Emphasize voluntariness, confidentiality, and that refusal or withdrawal will not affect the child’s care.

**•** Use the guide flexibly. Questions may be re-ordered, omitted, or expanded depending on the participant’s experience and emotional state.

**•** Use neutral probes such as “Could you tell me more about that?”, “What made you feel that way?”, and “Can you give an example?”

**•** If the participant has no previous research experience, use the brief hypothetical scenario at the end of this guide before asking the procedure-related questions.

# **Section A. Care-seeking context and understanding of research**

## **Q1. Please tell me about your child’s illness journey from the time the problem was first noticed until now.**

**Probes:** When was the child diagnosed?; Which hospitals did you visit?; Was there hospitalization, ICU care, or surgery?; How did the journey to the study center affect the family?

## **Q2. Where do you usually obtain health or treatment-related information for your child, and which sources do you trust most?**

**Probes:** Doctors or nurses; Hospital public account or website; Parent chat groups; Short-video or social media platforms; Family members or friends; Why do you trust some sources but not others?

## **Q3. How has the current care arrangement affected your family’s time, work, and finances?**

**Probes:** Transportation and accommodation costs; Wage loss or leave from work; Borrowing money or financial strain; Would reimbursement or travel support make a difference?

## **Q4. In your understanding, what is the difference between medical research and ordinary medical treatment?**

**Probes:** Have you ever heard about or participated in research before?; Do you think research is different from treatment?; Do you worry about being treated like a “test subject”?

## **Q5. What would make you feel more comfortable when considering participation in a research study?**

**Probes:** Hospital or department reputation; Recommendation from the treating doctor; Ethics approval and formal procedures; Clear explanation of risks and compensation; How the research team communicates

## **Q6. If research only involved questionnaires or observation, if it involved add-on sampling during routine blood collection, if it involved a swab, or if it required an extra needle puncture, how would you view each of these?**

**Probes:** For each procedure, ask whether it would be acceptable, uncertain, or unacceptable; What conditions would change the decision?; Would a better explanation or less pain make participation easier?

# **Section B. Participation trade-offs and family decision-making**

## **Q7. If the study only included non-invasive procedures or add-on sampling during routine care, how would you consider participation?**

**Probes:** Would it feel burdensome?; Would it interfere with treatment? Would the child be affected?

## **Q8. If the study required a mildly uncomfortable procedure such as a nasal or throat swab, how acceptable would that be?**

**Probes:** Would the child’s age or personality matter?; How would you help the child cooperate?; Would pain relief or distraction help?

## **Q9. If the study required one additional venipuncture for research, under what circumstances would you consider agreeing, and under what circumstances would you definitely refuse?**

**Probes:** Would clear necessity matter?; Would you prefer add-on sampling as an alternative?; Would a clear pain-management plan help?

## **Q10. In your family, who usually raises opinions first and who makes the final decision about whether to participate in research?**

**Probes:** Mother, father, grandparents, other relatives; Need for remote discussion with family members; Whether the child’s own view is considered

## **Q11. When is the best time to introduce a research study and ask for consent? When would be a bad time?**

**Probes:** During acute stress vs after stabilization; Outpatient clinic, bedside, discharge, or follow-up call; Need time to take materials home and think

## **Q12. If family members disagree, how is the disagreement usually resolved? What information would help the family reach a decision more easily?**

**Probes:** Need for a cooling-off period; Need for one-page summary, short video, or brief doctor endorsement

## **Q13. What is the most practical burden of taking part in research for your family?**

**Probes:** Extra time; Travel and accommodation; Work leave; Caregiving arrangements; Can procedures be aligned with routine follow-up?

# **Section C. Privacy, assent, and communication needs**

## **Q14. What concerns do you have about privacy, confidentiality, and data use in research?**

**Probes:** Who can access the data?; How long will it be stored?; Would secondary use need new permission?; Are genetic data especially sensitive?

## **Q15. If the study generated a result related to your child’s health, how and when would you want to receive that information?**

**Probes:** Through clinic visit, telephone, or WeChat; Who should explain it?; Would you want a written summary?

## **Q16. What do you think about the current requirement that children aged 8 years and older should also provide assent in addition to guardian consent?**

**Probes:** Does this feel reasonable?; What if the child refuses because of fear?; How should the family and doctors handle that situation?

## **Q17. Would explicit information about trial insurance, compensation, or institutional protection influence your decision about participation?**

**Probes:** What information would make you feel protected?; Would formal compensation language increase trust?

## **Q18. If researchers wanted to explain a study more clearly to families like yours, what type of communication would work best?**

**Probes:** One-page summary; Flow chart; Question-and-answer sheet; Short video or animation; Dialect or minority-language version

# **Section D. Recruitment optimization**

## **Q19. What could researchers do to make participation easier for families who travel from remote areas?**

**Probes:** Align procedures with routine clinical follow-up; Reduce waiting time; Avoid extra needle punctures; Provide travel support or reimbursement

## **Q20. Who should explain the study in order to make families feel most reassured?**

**Probes:** Research coordinator; Treating doctor; Department director; Combination of face-to-face explanation and later follow-up

## **Q21. What is the one thing you would most like researchers to improve so that families can make an informed decision without disrupting treatment?**

**Probes:** Message to other parents; Most important process change; Any final advice for the research team

# **Hypothetical scenario for participants with no prior research experience**

**Suggested script:** “Imagine that your child is receiving care at this hospital. A research study is being conducted without affecting treatment. It may involve questionnaires, non-invasive observation, a swab, or collection of a small extra sample during routine blood drawing. An additional needle puncture would only be proposed if necessary, and you could refuse without affecting treatment. Participation is voluntary, the information is de-identified, and you may withdraw at any time. The study may provide a small travel or time reimbursement.”

# **Closing**

**•** Briefly summarize the participant’s main concerns and priorities.

**•** Thank the participant for their time and contribution.

**•** Ask whether they would like to receive a general update about the study results and, if so, through which channel.
